# The Hereditary Hyperferritinemia-Cataract Syndrome in 2 Italian Families

**DOI:** 10.1155/2013/806034

**Published:** 2013-12-04

**Authors:** Katia Perruccio, Francesco Arcioni, Carla Cerri, Roberta La Starza, Donatella Romanelli, Ilaria Capolsini, Maurizio Caniglia

**Affiliations:** ^1^Pediatric Oncology and Hematology Section, Santa Maria della Misericordia Hospital, Località Sant'Andrea delle Fratte, 06156 Perugia, Italy; ^2^Haematology and Clinical Immunology Section, Department of Clinical and Experimental Medicine, Santa Maria della Misericordia Hospital, Perugia, Italy; ^3^ASL n. 1 Umbria, Italy

## Abstract

Two 8- and 9-year-old brothers were referred to the Pediatric Oncology Unit, Perugia General Hospital, because of hyperferritinemia. Both had a history of bilateral cataract and epilepsy. Genetic investigation revealed two distinct mutations in iron haemostasis genes; homozygosity for the HFE gene H63D mutation in the younger and heterozygosity in the elder. Both displayed heterozygosity for C33T mutation in the ferritin light chain iron response element. A 7-year-old boy from another family was referred to our unit because of hyperferritinemia. Genetic analyses did not reveal HFE gene mutations. Family history showed that his mother was also affected by hyperferritinemia without HFE gene mutations. Magnetic resonance imaging in the mother was positive for iron overload in the spleen. Cataract was diagnosed in mother and child. Further genetic investigation revealed the C29G mutation of the ferritin light chain iron response element. C33T and C29G mutations in the ferritin light chain iron response element underlie the Hereditary Hyperferritinemia-Cataract Syndrome (HHCS). The HFE gene H63D mutation underlies Hereditary Haemochromatosis (HH), which needs treatment to prevent organ damages by iron overload. HHCS was definitively diagnosed in all three children. HHCS is an autosomal dominant disease characterized by increased L-ferritin production. L-Ferritin aggregates accumulate preferentially in the lens, provoking bilateral cataract since childhood, as unique known organ damage. Epilepsy in one case and the spleen iron overload in another could suggest the misleading diagnosis of HH. Consequently, the differential diagnosis between alterations of iron storage system was essential, particularly in children, and required further genetic investigation.

## 1. Introduction

Hereditary Hyperferritinemia-Cataract Syndrome (HHCS), an autosomal dominant disease, is characterized by constitutively increased L-ferritin production, no iron overload, and bilateral cataracts [1]. Constitutive L-ferritin production is due to over 25 known point mutations in the L-ferritin gene iron responding element (IRE) on chromosome 19, altering IRE interactions with cytoplasmic iron regulatory proteins (IRP) [2, 3]. L-Ferritin protein aggregates, called inclusion bodies, accumulate preferentially in the lens [3, 4], provoking bilateral cataracts. This is the only known organ damage in HHCS [1–5].

Hereditary Haemochromatosis (HH) is an autosomal recessive disease which involves the iron storage system and is associated with HFE gene mutations on chromosome 6. The C282Y mutation is strongly associated with HH as about 90% of patients with iron overload have this genotype [6]. H63D, another frequent HFE gene mutation in Caucasians, underlies a predisposition to HH, but is associated with organ damage caused by iron overload only in the 30% of cases. Concomitant precipitating factors are adult age, comorbidities, and alcoholism or drug abuse [6, 7].

Here we describe members of two unrelated families with genetic disorders of the iron haemostasis system living in Umbria, Central Italy.

## 2. Case Report 1

Two 8- and 9-year-old brothers were admitted to hospital because of symptoms of epilepsy. Electroencephalograms (EEG) detected frontal night epilepsy in the older brother and Rolandic epilepsy in the younger (no other data are available). Magnetic resonance imaging (MRI) of the brain was normal in both children. Since routine blood test showed hyperferritinemia (1,380 and 1,461 ng/mL, resp., normal range: 11.0–307.0), with normal iron, transferrin, and haemoglobin levels, they were referred to our Pediatric Oncology and Haematology Unit. Both had bilateral generalized cataracts, diagnosed few years before. Genetic analysis showed that both carried the L-ferritin IRE gene [8] C33T mutation and the HFE gene H63D mutation. The latter was heterozygous in the older and homozygous in the younger. No heart or liver damage was detected. No treatment was given to either child. A watch-and-wait approach was adopted for cataract surgery and appropriate therapy for Rolandic seizures if they persisted. They both did not need any treatment for epilepsy to date.

**Figure 1:**
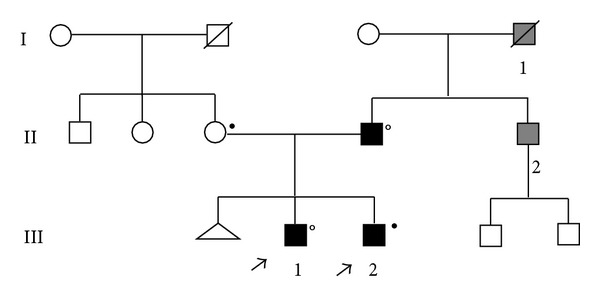
Pedigree of the first family with the presence of hyperferritinemia and cataract, because of the C33T mutation of the L-ferritin IRE region and H63D mutation of HFE gene. Black squares indicate affected individuals (II: 1; III: 1; III: 2) with early onset of cataract, hyperferritinemia, and L-ferritin IRE mutation. Grey squares indicate individuals (I: 1; II: 2) with early onset of cataract but unavailable for ferritin study and mutational analysis; °members heterozygous for HFE-H63D genotype; ^●^members homozygous for HFE-H63D genotype.

The children's mother was homozygous for the HFE gene H63D mutation, but she had normal ferritin values and no organ damage. The children's father had had cataract surgery at about 40 years of age. He carried the L-ferritin IRE gene [8] C33T mutation and was heterozygous for the HFE gene H63D mutation (Figure 1).

## 3. Case Report 2

A 7-year-old boy was referred to our unit because of hyperferritinemia (653 ng/dL). At clinical examination, we found that one lens had microopacities. HFE gene analyses were negative for known mutations. Further genetic investigation showed that the patient was heterozygous for the IRE C29G mutation of the L-ferritin gene [9].

**Figure 2:**
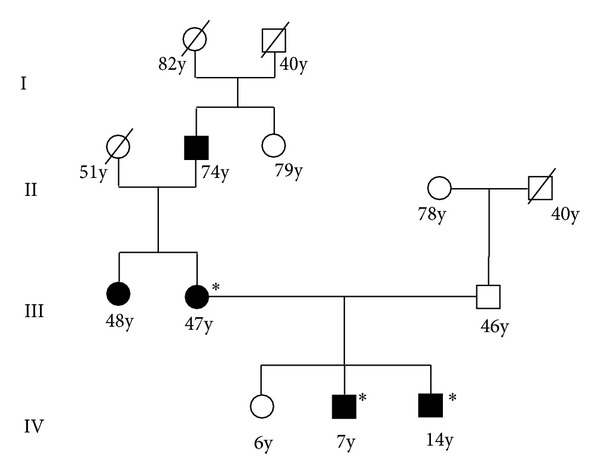
Pedigree of the second family with the presence of hyperferritinemia and cataract, because of the C29G mutation of the L-ferritin IRE region and H63D mutation of HFE gene. Black circles (female subjects) and squares (male subjects) indicated affected individuals (II: 1; III: 2; IV: 2) with early onset of cataract and hyperferritinemia; *members heterozygous for C29G IRE mutation. The others were unavailable for mutational analyses.

**Figure 3:**
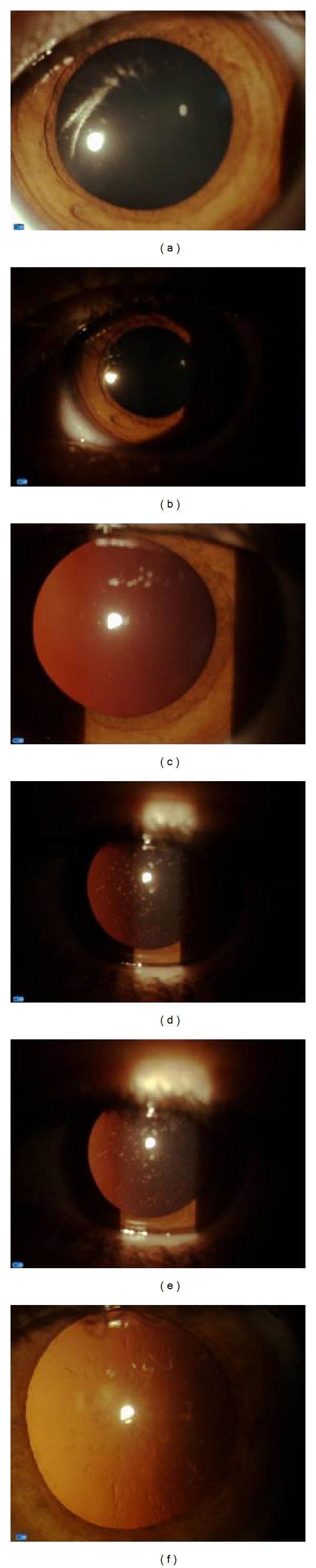
Progression of cataract in the left eye of the patient described in the second case report, from 2008 (a) to 2010 (b) to 2011 (c); bilateral cataract in the 14-year-old sister of the second case report: (d) right eye; (e) left eye. Cataract in the mother's eye of the second case report (f).

Family history showed that the patient's father and a 12-year old sister were healthy. A 14-year old sister was affected by generalized cataract and very high ferritin levels (1,796 ng/dL). His mother also had cataract associated with high ferritin levels (744 ng/dL) and was negative for HFE gene mutations, while MRI detected an iron overload in the spleen. Diagnosis showed HH, but phlebotomy led to severe anaemia and was definitively suspended after only two sessions. Further genetic investigation showed that not only the patient but also his mother and his 14-year-old sister were all heterozygous for the IRE C29G mutation of the L-ferritin gene (Figure 2). A watch-and-wait approach was adopted for all three while awaiting cataract surgery.  Figure 3 shows progression of cataract in the left eye of the child from 2008 to 2011 (Figure 3(a): 2008; Figure 3(b): 2010; Figure 3(c): 2011), bilateral cataract in the right Figure 3(d) eye and in the left Figure 3(e) eye of the 14-year-old sister and cataract in the mother's eye Figure 3(f).

## 4. Discussion

Hereditary Hyperferritinemia-Cataract Syndrome (HHCS) was diagnosed in all patients. The differential diagnosis was between HHCS, a relatively rare, autosomal dominant condition, and the autosomal recessive disorder HFE-related HH, one of the most common genetic diseases in Caucasians. Homozygosity for the C282Y mutation of the HFE gene on chromosome 6 is strongly associated with HH, as about 90% of patients with genetic iron overload have this genotype [6]. The H63D mutation of the HFE gene also underlies HH but is associated with iron overload-linked organ disease only in 30% of cases and usually in the presence of comorbidities, alcoholism, or drug abuse [6, 7].

In this report, HH was excluded in both families: in the first family because of heterozygosity in the elder brother, and age and lack of organ damage in the younger, even though he was a homozygotic carrier of the H63D mutation of HFE gene. It was excluded also in the second family because of the absence of any known mutation of HFE genes. We then focussed on HHCS, a rare, autosomal dominant disease with a prevalence of 1/200,000 [10]. HHCS main clinical features are elevated serum ferritin and early onset of bilateral cataracts. Transferrin saturation and iron are normal and indeed phlebotomy rapidly induces iron deficiency anaemia.

This is the first report of epilepsy and spleen iron overload, apparent signs of organ damage, in two separate, distinct families with HHCS. Mutations in the ferritin light chain IRE on chromosome 19 resulted in loss of ferritin synthesis inhibition. L-Ferritin protein aggregates accumulate preferentially in the lens [3, 4], provoking bilateral cataracts from childhood onwards. For this reason, bilateral cataracts in a child should alert paediatricians to the need for further tests.

Since both brothers in the first case carried H63D mutations and each was affected by a different form of epilepsy, we hypothesized that L-ferritin IRE mutations may play a role in neurological damage. One paper on an animal model reported increased ferritin expression in specific regions of the parahippocampal cortex in epileptic rats [11]. Neurological disorders have been reported with HH as iron may accumulate in the brain in late stage of disease usually after other organ damage, such as to the liver and heart.

In conclusion, the differential diagnosis between HHCS and HH is essential, particularly in children and young adults with cataract. Moreover, since HHCS is very rare, paediatricians should report all other symptoms besides cataract that occur in association with it, so as to further understand the physiopathology of this uncommon disease.

## Abbreviations

HFE: HH: Hereditary Haemochromatosis
HHCS: Hereditary Hyperferritinemia-Cataract Syndrome
IRE: Iron responding element
MR: Magnetic resonance.
